# Can 18-Month-Olds Revise Attributed Beliefs?

**DOI:** 10.1162/opmi_a_00087

**Published:** 2023-07-21

**Authors:** Ildikó Király, Katalin Oláh, Ágnes M. Kovács

**Affiliations:** MTA-ELTE Social Minds Research Group, Psychology Institute, Eötvös Loránd University, Budapest, Hungary; Department of Cognitive Science, Central European University, Budapest, Hungary

**Keywords:** theory of mind, episodic memory, prospective and retrospective processes in mindreading, memory development

## Abstract

Successful social interactions rely on flexibly tracking and revising others’ beliefs. These can be revised prospectively, new events leading to new beliefs, or retrospectively, when realizing that an attribution may have been incorrect. However, whether infants are capable of such belief revisions is an open question. We tested whether 18-month-olds can revise an attributed FB into a TB when they learn that a person may have witnessed an event that they initially thought she could not see. Infants first observed Experimenter 1 (E1) hiding two objects into two boxes. Then E1 left the room, and the locations of the objects were swapped. Infants then accompanied Experimenter 2 (E2) to the adjacent room. In the FB-revised-to-TB condition, infants observed E1 peeking into the experimental room through a one-way mirror, whereas in the FB-stays-FB condition, they observed E1 reading a book. After returning to the experimental room E1 requested an object by pointing to one of the boxes. In the FB-stays-FB condition, most infants chose the non-referred box, congruently with the agent’s FB. However, in the FB-revised-to-TB condition, most infants chose the other, referred box. Thus, 18-month-olds revised an already attributed FB after receiving evidence that this attribution might have been wrong.

## INTRODUCTION

In everyday life, humans continuously engage in social interactions, they communicate, coordinate, collaborate or even compete with each other. These activities require tracking others’ mental states adequately (Byom & Mutlu, 2013; Boyd & Richerson, 1996) in situations where the environment is constantly changing, and one’s own beliefs, as well as those attributed to others, have to be frequently updated. Thus, the mindreading system must be highly effective and dynamically adaptive to the needs of such a dense social milieu, raising the question of how and when flexible mindreading is achieved in ontogeny. Here we aim to investigate whether already infants can revise an already ascribed belief in the face of new and relevant information.

Beliefs can be attributed to others in at least two different ways: *prospectively*, and *retrospectively* (Király et al., 2018). Prospective belief tracking refers to the processes via which an observer tracks what another person can and cannot witness of the unfolding events and uses this as a basis to infer which representations the other may form. In such cases, as soon as there is a change in the situation, which should bring about a belief change in the observed agent, the observer performs the adequate inferences and updates in a prospective manner, from infancy onwards (Ganea & Harris, 2010; Kovács, 2016; Song et al., 2008; Schulze & Buttelmann, 2022; Tauzin & Gergely, 2018). For instance, in the classical Sally Anne task, after attributing a false belief to Sally (e.g., that the marble is in the basket), if she later happens to look into the box and sees her marble there, she must update her belief. Based on the directly available evidence that has a causal role in forming a new belief (seeing leads to knowing), the observer will attribute a new belief to Sally.

Conversely, retrospective processes are recruited when, based on some new information, it becomes likely that a specific belief might have been incorrectly attributed, and consequently, it must be revised, (similarly to how the backtrack re-evaluation of causal relations takes place, Gerstenberg et al., 2013, or how we modify our earlier inferences in other domains). As an example of how we re-evaluate our own beliefs based on retrieving earlier events, imagine you are a fire safety representative and one day the fire alarm goes on. You first arrive to the conclusion that all present colleagues know about it and have safely left the building. However, afterwards you realize that someone could have been working in the soundproof booth at the time of the alarm. To decide whether there may be such a person, you will rely on your memory and try to recall all the people you met on the way out, to figure out whether someone is missing.

In fact, retrospective, memory-based processes require the observer to recall and take into account information not previously considered, and use the novel piece of information for modifying one’s own description and inference of the original situation (Klein et al., 2009).

A study by Király et al. (2018) aimed to disentangle the contribution of prospective and retrospective processes in the belief attribution of children. In this study, after hiding two objects into two boxes, the experimenter put on a pair of sunglasses, and the locations of the objects were swapped by another person. Then, the experimenter left, and participants could explore her sunglasses, which turned out to be either opaque or transparent. In the test, the experimenter returned, pointed to one of the boxes and asked for an object. After learning that the sunglasses were opaque, children could retrospectively revise the experimenter’s belief from ‘true’ to ‘false’ and (re)-compute its content concerning the location of the objects. In the condition where the sunglasses were transparent, retrospective revision was not necessary. The results suggested that 36-month-olds, but not 18-month-olds, responded differently in the two conditions, only the older age group showing evidence for retrospectively attributing a false belief (Király et al., 2018).

However, such attributions could happen either by (i) revising an already (prospectively) attributed TB into a FB, or by (ii) computing the FB of the experimenter triggered by the novel piece of information fully retrospectively, without having computed a true belief earlier. Importantly, these two alternatives are different not only in the involved processes, but invite different explanations for why 18-month-olds might have failed in this task.

In particular, according to the first alternative (i), that relies on revising a prospectively attributed belief, young children may mainly use the prospective route for attributing beliefs to others, and retrospective revisions may pose a challenge. Thus, a belief is attributed as the events unfold (e.g., the agent seems to have visual access to the location swap), and it is maintained to serve future predictions or interactions (e.g., to interpret the protagonist’s requests for an object). The prospective route involves belief ascriptions that are based on the access of the protagonist to specific information (what the protagonist is aware of). Consequently, retrospective update processes should operate on these already attributed beliefs. When the observer realizes that the sunglasses worn by the protagonist were opaque (and remembers that she wore those sunglasses at the moment of the swap), a retrospective revision of her belief is initiated. The prospectively attributed, but unwarranted true belief is identified (‘she believes object A is in the box on the left’) and discarded, and by the retrieval of the original episode (the event she could last see) the new belief is computed (‘she believes object A is in the box on the right’), via retrospective revision.

Alternatively, one may argue for the second alternative (ii) that such situations do not trigger retrospective revisions but retrospective attributions, in case true beliefs were not computed in the task. One could argue that we attribute beliefs to others only when a perspective divergence is detected. For instance, in the Sally Anne task, when Sally leaves the scene and the object’s location changes, this event triggers not only an update of one’s own belief about the state-of-affairs, but one may ascribe a divergent belief to Sally at this point based on what she has witnessed earlier. Importantly, an initial true belief ascription is not necessary in this process. When there is no divergence, one may simply tag one’s own representation as shared with another person (see Martin & Santos, 2016), or encode this congruency as part of common knowledge. Thus, if no belief was ascribed, no belief can be revised, but specific events may nevertheless trigger retrospective attributions. In the task of Király et al. (2018) when children learned that the sunglasses were opaque, this could have induced a search in memory regarding how this information could alter the agent’s perception of the events (i.e. not seeing the location change) and consequently her beliefs.

In light of these two alternatives, 18-month-olds might have failed the task of Király et al. (2018) either because they could not revise an already attributed true belief (failure of retrospective belief *revision*), or because of difficulties in computing a belief retrospectively upon receiving new, belief-relevant information (failure of retrospective belief *attribution*), if true beliefs were not computed earlier. Thus, it is still unclear whether 18-month-olds can *revise* already attributed beliefs at all, as the earlier used tasks may not necessarily mandate the attribution of true beliefs (see recent debates in Phillips et al., 2021 and related commentaries, Dudley & Kovács, 2021).

To directly address this question, we target scenarios where relying on common knowledge is not sufficient, and infants have to first attribute beliefs to others (i.e. when attributing divergent or false beliefs), in order to investigate whether they can later flexibly revise these. Evidence from different tasks seems to suggest that by 18 months, infants are able to attribute false beliefs via monitoring the perceptual access of the protagonist (Király et al., 2018; Knudsen & Liszkowski, 2012; Kovacs et al., 2010; Scott & Baillargeon, 2017; Senju et al., 2011; Southgate et al., 2010; but see also replication problems in Schuwerk et al., 2018; Crivello & Poulin-Dubois, 2018).

In the present task, successful performance would necessarily require updating an attributed belief: infants will need to revise an *already attributed false belief* upon learning that the agent could have witnessed a situation that they initially thought had not been perceived by her. While 3-year-olds can retrospectively update attributed beliefs in such tasks (Király et al., 2018, Exp. 3), the question of whether 18-month-olds can revise their attributed false beliefs was not directly tested before.

In the current study, 18-month-olds first observe two novel objects hidden by Experimenter 1 (E1) into two boxes. Then E1 leaves the room, and the locations of the objects are swapped. Infants are then asked to accompany Experimenter 2 (E2) to the neighboring room to call E1 back. When they enter the room, infants in the FB-revised-to-TB condition, observe E1 peeking into the experimental room through a one-way mirror. In in the FB-stays-FB condition, E2 is reading a book and the one-way mirror is covered. In a third condition (FB condition), infants do not leave the experimental room and receive no extra information. After returning to the room, E1 requests an object by pointing to one of the two boxes. In line with previous studies (Király et al., 2018; Southgate et al., 2010), we expect infants to interpret the gesture as referring to the object which the experimenter believes to be inside, thus this referent mapping is dependent on the attributed belief. If infants attribute false beliefs, they should choose the non-referred box, given that the experimenter was not present when the location of the objects was swapped. Furthermore, if they can revise a false belief into a true belief after seeing E1 peeking through a one-way mirror, they are expected to choose the referred box.

## METHODS

### Participants

Sixty 18-month-old infants were recruited (20 per condition). 12 children were excluded because of experimenter error (2 per condition) or quitting the experiment before the test phase (FB:2; FB-revised-to-TB:1; FB-stays-FB:1), or choosing both objects during the test phase (FB-revised-to-TB: 2). The final sample was 15 infants in the FB-revised-to-TB condition; 16 infants in the FB condition, and 17 infants in the FB-stays-FB condition (mean age = 18.12 months, range: 17.5–18.5 month).

### Procedure

The procedure and sample size selection were based on Király et al. (2018) Experiment 3. Warm up trials preceded the testing phase (see details in supplemental materials, https://osf.io/7jvpf) and test trials had three phases.

#### Belief Induction Phase.

E1 first gave the infants the two novel objects to explore for about 10 seconds. These objects were not labelled in this phase. E1 then placed an object in each box and closed the lids. The location of the objects was counterbalanced across infants. At this point, Experimenter 2 (E2) asked E1 to go out for a while. After E1 left, E2 deceptively approached the boxes, switched the objects, and closed the boxes. This phase was identical for all three conditions.

#### Belief Revision Phase.

In the FB condition, replicating Southgate et al. (2010), infants stayed in the room, while E2 went to call back E1 alone. In the FB-revised-to-TB and FB-stays-FB conditions all infants accompanied E2 to the adjacent room to call E1 back. In FB-revised-to-TB condition, when they entered the adjacent room, infants saw E1 peeking into the experimental room through a one-way mirror. Despite the arrival of E2 and the child, the experimenter continued looking through the one-way mirror and did not interact with them. E2 encouraged the child to look through the one-way mirror (all children looked through it). Note that during all this time, the objects were not visible (were in the closed boxes). In the FB-stays-FB condition, when they entered the adjacent room, infants saw E1 sitting on a chair and reading a book, while the one-way mirror was covered with a curtain. Here too, despite E2 and the child being there, the experimenter continued reading and did not interact with them. After being away approximately 45 seconds, E2 asked E1 and the child to return to the experimental room in both conditions, without any further interaction.

#### Test-phase.

**Figure 1.**
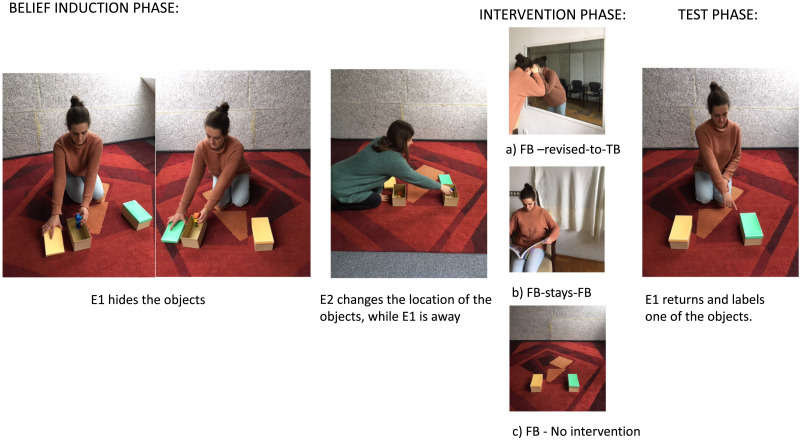
**The schema of the experimental design.** I. Belief induction phase: E1 puts two objects into two different boxes. After this, in her absence, E2 swaps their locations. II. Intervention phase: children accompany E2 to call E1 back to the room, and children find E1 in the other room a) looking through the one way mirror (FB revised to TB condition); b) reading a book (FB stays FB condition). In the third condition c) children stay in the room and wait till E1 returns (FB-No intervention). III. Test phase: E1 points to a box and labels one object, then asks for that object via naming it while holding both boxes towards the child.

After returning E1 greeted the infant, and sat behind the two boxes. E1 then pointed at one of the boxes (counterbalanced across infants) and said (in Hungarian), “Do you remember what I put here? I put a sefo here. Shall we play with the sefo?”, alternating gaze between the infant and the referred box twice. E1 then grasped both boxes, extended her arms towards the child and simultaneously opened the lids of both boxes that were oriented towards the child. At this point, the contents of the boxes became visible only to the child. E1 then said, “Can you give me the sefo?”, while looking directly at the child, and not looking towards either box. E1 repeated the question until the child began to approach one of the boxes, pointed towards one of the boxes, or until 180 seconds had passed (see Figure 1).

### Coding

The sessions were video-recorded and coded off-line. The dependent measure was the choice that children made in response to E1’s request. The first response towards one of the boxes, after E1 had said, ‘Can you give me the sefo?’ was coded as the child’s choice and was categorized as choosing the referred or the non-referred box. Both reaching and pointing responses were accepted as valid choices. All sessions were coded also by a second observer, blind to the experimental condition. Interrater agreement was 97% (Cohen’s Kappa: 0.933).

## RESULTS

**Figure 2.**
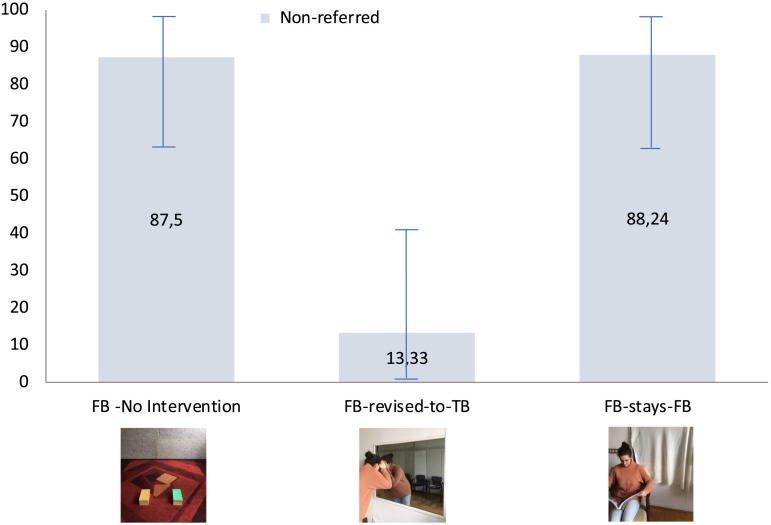
The proportion of 18-month-old infants choosing the non-referred box (as opposed to the referred one) in the three conditions with 95% bootstrapped confidence intervals.

In the FB condition 14 participants (87,5%) chose the non-referred box and 2 (12,5%) chose the referred one. In the FB-stays-FB condition which includes an intervention phase with no belief relevant elements, 15 infants (88,24%) chose the non-referred box, and 2 (11,76%) the referred one. However, importantly, in the FB-revised-to-TB condition, in which children witnessed E1 peeking through the one-way mirror, infants showed the expected opposite pattern, 13 infants (86,66 %) chose the referred box, and only 2 of them (13,33%) chose the non-referred one (see Figure 2, data is available at: https://osf.io/c9zs5.

A 2 × 3 chi-square test revealed a difference in the pattern of choices between the three conditions (chi-square = 25,1, df = 2, *p* = .00001). Fisher’s exact tests confirmed that the number of infants choosing the referred box differed significantly between the FB-revised-to-TB and FB-stays-FB conditions (*p* = .00003; OR: 48.75), and also between the FB-revised-to-TB and FB conditions (*p* = .00005, OR: 45.5), while FB and FB-stays-FB conditions did not differ from each other.

Results in the FB condition replicated the results of Southgate et al. (2010) as infants chose the non-referred box above chance level (binomial test, *p* = .0018). Regarding the FB-stays-FB condition, a binomial test similarly yielded that infants chose the non-referred box above chance level (*p* = .001) providing yet a further conceptual replication that infants take into account the experimenter’s false belief when disambiguating the referent in the two conditions. Importantly, in the FB-revised-to-TB condition, infants chose the referred box significantly above chance level (*p* = .003), providing clear evidence for retrospective belief revision at such an early age.

## DISCUSSION

### False Belief Attribution and Retrospective Revision in 18-Month-Olds

Across three conditions, we found that infants as young as 18 months can not only track others’ false beliefs to identify the correct referent in a communicative context, but when new information comes in that warrants retrospective belief revision, they can also flexibly update these attributed beliefs.

Flexibly monitoring the mental states of communicative partners is essential for successful and rapid information exchange in social interactions (Sperber & Wilson, 2002). This could be established by prospective belief attributions based on fast mapping information the interaction partner has direct access to, or via retrospective attributions relying on revising an already maintained belief in light of indirect evidence.

While previous studies suggested that 18-mo-olds can use the prospectively attributed belief content of a communicator for referent disambiguation (Southgate et al., 2010; Knudsen & Liszkowski, 2012), and served as evidence for the early use of belief ascription in social interactions, the robustness and replicability of these results is disputed (see Grosse Wiesmann et al., 2017; Dörrenberg et al., 2018 for non-replication and Király et al., 2018 for replication). The FB condition of the present experiment successfully replicated the results of Southgate et al. (2010) (direct replication) and serves as a conceptual replication of the FB-FB condition of Király et al. (2018). Furthermore, data from the current FB-stays-FB condition also counts as an additional conceptual replication of these experiments. These together support the proposal that 18-month-olds prospectively attribute false beliefs to communicative partners.

Importantly, results from the crucial condition of the current study, the FB-revised-to-TB condition, suggest that 18-month-olds could *retrospectively revise* an already attributed belief. They revised a false belief into a true belief in a situation in which they first assumed that the communicator had no perceptual access to the critical events, as she was not present, and thus first attributed a false belief. Crucially, upon encountering new information that warranted belief revision (realizing that E1 could see the events through the window from the other room), children inferred that, although she was not present in the room, she did have perceptual access to the critical events and successfully revised her earlier computed belief. A recent study of Liszkai-Peres et al. (2021) found an analogous pattern of mnemonic competence in 2-year-old children: they appropriately recalled playful goal directed actions (e.g., reaching for an object with a long wooden spoon) after a week delay, and they were also able to revise their strategies of goal attainment in a changed context. In particular, they computed the relevance of the tool use given specific environmental constraints (when the objects were far), and switched to a simpler solution when it became clear that it was no longer relevant (when objects were close). These updating processes likely rely on infants’ developing working memory capacities, which seem reliable already in the second year of life (Cheng et al., 2019), allowing infants to retrieve multi-step (3 component) event sequences for complex planning (Blankenship & Kibbe, 2022).

### Potential Mechanisms Supporting Belief Revision

While retrospective belief revision is rather impressive at such a young age, it is still an open question which mechanisms may support this process. At minimum, belief revision should (i) necessarily operate on actively maintained belief contents, and (ii) relate new information to already encoded contents. These seem mandatory in triggering the process of revision, namely that one should recognize whether the observed novel information (i.e., that the other person could peek into the room) is relevant for the attributed belief and if so, a revision should be applied.

Critically, as mentioned earlier, retrospective revisions should necessarily operate on *a specific* belief content in the light of new evidence (i + ii). This requires forming a link between two belief-relevant events—that the protagonist did not see the location change and that it was later discovered she could see through the one-way mirror—as there can be no revision without making a connection to a past event. Thus, we conjecture that to allow for retrospective revisions, attributed belief representations should include some information about their origins, specifically about what serves as a basis for forming the belief in the first place. In the current task, one could initially encode that the agent did not see the location change, which results in a specific belief, based on what she has seen earlier. Thus, when later belief-relevant information is encountered (one learns that the protagonist could see through the one-way mirror), this will be in conflict with the premise based on which the false belief was attributed (‘not seeing event B, therefore believing A’). If this premise is stored together with the belief content and marked as invalid, this should render the belief unjustified, triggering the need for a revision. Thus, a successful precondition for retrospective revision is identifying that a belief is unjustified and discarding it.

However, to be able to respond flexibly to a given situation, besides discarding the initial belief content, one should be able to revise the attributed belief with new, justified content. Finding the adequate new belief content that replaces the old one could be realized via recruiting at least two different kinds of mechanisms.

According to the first possibility, termed here ‘*Retrospective revision via reverting to common knowledge*’, after the revision process has started, and the initial belief has been identified as unwarranted and is discarded, the new belief content is defined via substituting the old one with the observer’s own knowledge. For instance, realizing that the partner could have seen everything that the observer has seen recently, could result in the recognition that the model has the same knowledge about reality. Therefore, the related first person representation—about the actual location of the objects—is selected.

According to a second possibility, termed here ‘*Retrospective revision via (true) belief computation*’, after the initial belief was identified and discarded, the new belief content is defined by computing and attributing a new (true) belief. Based on the updated visual access of the interlocutor regarding the past event, one computes that they have a true belief about the location of the objects.

One might wonder how to adjudicate between these possibilities, given the challenges of distinguishing between the attribution of knowledge and/or true beliefs (Phillips et al., 2021). Based on parsimony arguments, one might suggest that the mechanisms recruited by the first alternative may seem simpler in terms of their representational requirements, as there is no need to recollect the original episode to compute the belief content of the partner. Rather, encoding the information monitored in the situation (first person knowledge) as shared may be enough for the success of revision. In contrast, the second alternative would hypothesize that in order to identify the actual true belief in the situation, the emerging task for infants would be to *compute a new belief retrospectively*, which would necessitate relying on episodic memory processes. Thus, in terms of the contribution of memory processes, while both alternatives require the tracking of the sources of (false) beliefs, the second alternative—which is an actual retrospective attribution process—requires the accurate use of episodic memory capacities as well (Mahr & Csibra, 2018) to retrieve what event details might be relevant given the updated perceptual access information. In either case, independent from what mechanisms are recruited to fill in the new belief content, the results suggest that infants are able to monitor the validity of already attributed beliefs, and discard them appropriately and replace them with a new content.

In light of the present findings, one might argue that the failure of 18-month-olds in the study of Király et al. (2018) could have been due to the difficulties in ascribing a belief retrospectively, given that infants likely did not attribute a true belief that could be later revised, and not due to the lack of the ability to *revise* itself, as the present study provides evidence for flexible belief revision. Indeed, there is no reason to suppose that the revision of a false belief would be easier in the present task than that of a true belief in Király et al. (2018). This capacity constraint may be similar to that of found by Liszkai-Peres et al. (2021) in a different domain regarding the re-computation of the relevance of tool-use in a novel context. Two-year-old children could recall the tool use when it was first relevant and remained relevant, and even revised its relevance when it turned irrelevant in the novel context. However, they did not recall the tool use when it was originally irrelevant, but it turned to be relevant in the novel context, suggesting that given that relevance was not computed earlier, they could not revise it and a retrospective re-assignment of relevance did not take place.

One might wonder whether there are alternative accounts that might explain our findings. One could propose that infants, after inferring that the protagonist could have seen everything, became confused, and consequently, abandon any attribution. According to this explanation, infants would decide to follow the pointing gesture with fidelity because they are uncertain about what E1 might believe. However, please note that this ‘confusion’ was triggered by a very specific event, which resulted in discarding an earlier attributed false belief just as described above, recruiting revision mechanisms that necessarily operate on a specific belief content in light of new evidence. Importantly, this possible ‘confusion’ was specific to the condition where the model was peeking into the room, and not the result of an intervening event in general—i.e., going to the adjacent room. The necessary implication is then that the disruption was caused by the interpretation of the observed event, namely, inferring that the model might have seen everything, and that the earlier computed false belief must be discarded. Thus, even this alternative account in which infants may be uncertain whether the experimenter could see the events, seems to support infants’ capacity for (i) attributing a false belief and (ii) realizing when it is adequate to discard it.

In sum, the current findings suggest that infants engage in complex retrospective belief revision processes, that entail attribution, maintenance and even some justification of beliefs, and if found to be unjustified, their discarding. Future research should uncover whether this basic tracking of the sources and the justification of beliefs, specifically the (lack of) perceptual access of the partner, could be seen as the prerequisite of source memory (Kampis et al., 2018). The present data support the view that early belief attribution is flexible, and retrospective belief revision operates early on, which opens up the possibility for social coordination based on context sensitive and quick inferences about others’ mental states.

## ACKNOWLEDGMENTS

This study was supported by the Momentum Program of the Hungarian Academy of Sciences to I. Király (LP-2017-17/2017). Partial funding came from James S. McDonnell Foundation, Grant n° 220020449. We thank parents and children participating in this study and Baross, J. and Hegedűs A. for their help with data collection, and György Gergely for his valuable comments on the manuscript. Special thanks to the Paris Institute of Advanced Studies, where the first author was a Resident Fellow during writing up this paper.

## DATA AVAILABILITY STATEMENT

The data that support the findings of this study are openly available in OSF at: https://osf.io/c9zs5/.

## FUNDING INFORMATION

This study was supported by the Momentum Program of the Hungarian Academy of Sciences (LP-2017-17/2017). Partial funding came from the James S. McDonnell Foundation, Grant n° 220020449.

## CONFLICT OF INTEREST DISCLOSURE

The authors are in full consensus on the content of the manuscript and the ordering of authors. All co-authors contributed to the present manuscript substantially, and will be informed about any decisions regarding the manuscript by the corresponding author. There are no conflicts of interests.

## ETHICS APPROVAL STATEMENT

All the experiments presented in the paper were approved by the ethical committee of the University (Ethical Committee of the Eötvös Loránd University, Budapest, approval No.: 2018/126-2).
